# Ambient particulate matter exposure and cardiovascular diseases: a focus on progenitor and stem cells

**DOI:** 10.1111/jcmm.12822

**Published:** 2016-03-14

**Authors:** Yuqi Cui, Qinghua Sun, Zhenguo Liu

**Affiliations:** ^1^ Dorothy M. Davis Heart and Lung Research InstituteDivision of Cardiovascular MedicineThe Ohio State University Wexner Medical CenterColumbusOHUSA

**Keywords:** air pollution, PM, cardiovascular disease, stem cell, endothelial progenitor cell

## Abstract

Air pollution is a major challenge to public health. Ambient fine particulate matter (PM) is the key component for air pollution, and associated with significant mortality. The majority of the mortality following PM exposure is related to cardiovascular diseases. However, the mechanisms for the adverse effects of PM exposure on cardiovascular system remain largely unknown and under active investigation. Endothelial dysfunction or injury is considered one of the major factors that contribute to the development of cardiovascular diseases such as atherosclerosis and coronary heart disease. Endothelial progenitor cells (EPCs) play a critical role in maintaining the structural and functional integrity of vasculature. Particulate matter exposure significantly suppressed the number and function of EPCs in animals and humans. However, the mechanisms for the detrimental effects of PM on EPCs remain to be fully defined. One of the important mechanisms might be related to increased level of reactive oxygen species (ROS) and inflammation. Bone marrow (BM) is a major source of EPCs. Thus, the number and function of EPCs could be intimately associated with the population and functional status of stem cells (SCs) in the BM. Bone marrow stem cells and other SCs have the potential for cardiovascular regeneration and repair. The present review is focused on summarizing the detrimental effects of PM exposure on EPCs and SCs, and potential mechanisms including ROS formation as well as clinical implications.

## Introduction

Air pollution is a major challenge to public health. Ambient particulate matter (PM) is the key component for air pollution. A recent Global Burden of Disease Study showed that PM exposure is responsible for 3.2 million deaths per year and 76 million years of healthy life lost 1. Particulate matter exposure could be especially a major health problem in the developing countries as the fine PM levels in some developing countries are reported to be 10 times higher than that in the developed countries 2. The majority of mortality following fine PM exposure has been related to cardiovascular diseases 1.

Particulate matter is a mixture of various particles including metals, crustal material and bio‐aerosols 3, 4. The particles with a median aerodynamic diameter of <2.5 μm (PM_2.5_) and <10 μm (PM_10_) are of serious global health concerns because of their close association (especially PM_2.5_) with the detrimental effects of air pollution on our health 5, 6. The PM_2.5_ exposure has been reported to produce a variety of deleterious effects on cardiovascular system including (but not limited to) vascular dysfunction, reduced heart rate variability and enhanced risk for thrombosis 3, 7. Long‐term exposure to PM_2.5_ has been demonstrated to accelerate the process of atherosclerosis and vascular inflammation in apolipoprotein E^−/−^ mice with high‐fat diet 8, and increase blood pressure in human and animal models 9, while short‐term exposure to PM_2.5_ could also induce hypertension 10.

Endothelial dysfunction or injury is considered one of the major factors that contribute to the development of atherosclerosis and coronary heart disease 11, 12. Endothelial progenitor cells (EPCs) play a critical role in vascular re‐endothelialization, angiogenesis and prevention of neointima formation after vascular injury 13, 14, 15, 16. The number and function of stem cells (SCs) and EPCs are significantly decreased in the animals exposed to PM_2.5‐10_. However, the mechanism(s) for PM_2.5‐10_ exposure‐induced impairment of EPCs is not fully understood. Current data strongly support the concept that the effect of PM‐exposure on EPCs might be related to increased level of reactive oxygen species (ROS) and inflammation 17, 18, 19, 20. Accumulating pre‐clinical and clinical data suggest that bone marrow stem cells (BMSCs) and other SCs could significantly contribute to cardiovascular regeneration and repair after damage like myocardial infarction and myocarditis. Exposure to ambient PM related to air pollution is a constant lifelong hazard for all of us. It is therefore important to summarize the adverse consequence of PM exposure on human participants. The present review is focused on the impact of PM exposure on cardiovascular system with special efforts on progenitor cells and SCs as well as related mechanisms especially ROS formation.

## PM and cardiovascular diseases

It has been demonstrated that PM_2.5_ exposure could induce various cardiovascular diseases including atherosclerosis, hypertension, stroke and Type 2 diabetes mellitus (DM) 21. American Heart Association and the Environmental Protection Agency have officially acknowledged the detrimental effects of PM_2.5_ on cardiovascular system and related morbidity outcome 2. The Harvard Six Cities study showed that the cardiopulmonary mortality was increased up to 37% in the population exposed to high levels of ambient PM_2.5_ over a period of 14–16 years 22. The analysis from a population of 50 million living in the major U.S. cities (The National Morbidity, Mortality and Air Pollution Study) indicated that an increase of 10 μg/m^3^ in PM_10_ was related to an increase in 0.68% in cardiopulmonary mortality 23, 24, 25. Every 10 μg/m^3^ increase in PM_2.5_ exposure was also associated with an increase in 4.5% in coronary artery disease (CAD) 26. Conversely, it was estimated that each 10 μg/m^3^ decrease in PM_2.5_ was associated with an increase in 0.61 years in mean life expectancy in the United States 27. In addition, there was a close relationship between NO_2_ and PM_2.5_ and the risk of acute myocardial infarction and hospitalization in the U.S 28, 29. Similarly, the Air Pollution and Health European Approach study analysed a population of 43 million in 29 large European cities, and showed that PM_10_ was closely related to cardiovascular diseases 30.

Particulate matter pollution was also correlated with a significant increase in blood pressure. It has been reported that there was a 2.8 mmHg increase in systolic blood pressure and 2.7 mmHg increase in diastolic blood pressure in patients in Boston over 5 days for every 10.5 μg/m^3^ increase in PM_2.5_ levels 31. Similarly, studies have shown that increased PM_2.5_ levels were associated with mean increases in systolic blood pressure of 3.2 mmHg in Detroit, Michigan, USA 32. In addition, it was observed that a significant rise in diastolic blood pressure (6 mmHg) in 23 normotensive patients after a 2‐hr exposure to PM_2.5_ and O_3_ compared with the patients without exposure 33. These observations support the conclusion that there is indeed a close association between increased blood pressure and PM_2.5_ exposure in human participants 34.

Air pollution has been shown to increase the risk for obesity, hypertension, chronic pulmonary disease and cardiovascular disease in the elderly 35, 36. Long‐term exposure to PM_2.5_ could induce insulin resistance and mitochondrial alteration in adipose tissue 37, thus further causing or exaggerating DM 38, 39. These studies provide additional evidence that PM exposure is directly associated with cardiovascular diseases, and also closely related with conditions like DM directly associated with increased cardiovascular morbidity and mortality.

## Progenitor cells and cardiovascular diseases

Endothelial dysfunction or injury is considered one of the major factors that contribute to the development of cardiovascular diseases like atherosclerosis 11, coronary heart disease 12, congestive heart failure 40, 41, 42 and periphery artery disease 43. Bone marrow‐derived EPCs play a critical role in vascular re‐endothelialization, angiogenesis and prevention of neointima formation after vascular injury 13, 15, 44. There is an obvious inverse relation between the level of circulating EPCs and the risk of cardiovascular events in the patients with angiographically documented CAD 45. Similarly, impaired function of EPCs such as deficiency in migratory response and poor angiogenic capability has a negative correlation with the severity of CAD 46. The important role of EPCs in maintaining the structural and functional integrity of the blood vessels has been well‐established and extensively discussed in many excellent reviews 47, 48. Thus, the level of circulating EPCs has been an important and independent predictor for cardiovascular outcome in CAD patients 45, and it is crucial to preserve the number and function of EPCs at a healthy level for the normal functionality of vasculature in patients with cardiovascular diseases. A variety of factors are critically involved in the regulation of the *in vivo* dynamics of EPC number and function, including (but not limited to) cytokines and growth factors like granulocyte‐stimulating colony stimulating factor and VEGF 40, 41, 42, nitric oxide, pharmacological agents like statins 49 and environmental factors like air pollution 19, 50. Some disease states like hyperlipidaemia, DM, inflammation, oxidative stress, ischaemia and chronic heart failure are also important for the dynamic changes of EPCs *in vivo* 19, 51, 52, 53, 54.

It is important to point out that the identification and characterization of EPCs have been very challenging and complex, and even controversial as excellently summarized in a few recent review articles 55, 56, 57. There are currently no unified criteria to define EPCs as yet. Therefore, the terminology ‘EPC’ was adopted from the original papers without modification to preserve the originality. The obvious limitation or confusion was that ‘EPCs’ from different studies might not be the same cell populations with different with cell markers in the literature. There are also multiple sources for circulating EPCs, including BM and non‐BM origins such as liver and spleen 58, 59. The number and function of circulating EPCs could be delicately determined by the combined outcome of EPC mobilization, differentiation, proliferation and apoptosis at sites of different sources. Accumulating evidence from pre‐clinical and clinical studies suggests that cell‐based therapy with progenitor cells (such as EPCs, CD34^+^ cells, c‐kit+ cells and adipose tissue progenitor cells, APCs) and SCs (including BMSCs) remains an attractive option for tissue regeneration and repair after significant damages like myocardial infarction and myocarditis or stroke 60, 61, 62. To achieve the optimal outcome for cell‐based therapy, the quality of the cells needs to be preserved both in the donors and in the recipients before and after the *in vivo* delivery. It is well known that only a small fraction of cells could survive after *in vivo* delivery (both locally and systematically) 63, 64. However, very little is known on how the quality (including the number and function as well as differentiation potential) of the progenitor cells and SCs could be affected by the potential factors *in vivo*.

## PM and progenitor cells

Epidemiological and experimental studies have shown that there is an obvious relationship between exposure to airborne pollutants and poor cardiovascular health 50. Although very limited data are available on the mechanisms for air pollution‐related cardiovascular diseases, induction of endothelial dysfunction by PM_2.5_ (not able to be filtered by the respiratory tract) is believed to be one of the mechanisms for the adverse effects of air pollution on cardiovascular system in a population‐based study with children and adolescents 65.

**Figure 1:**
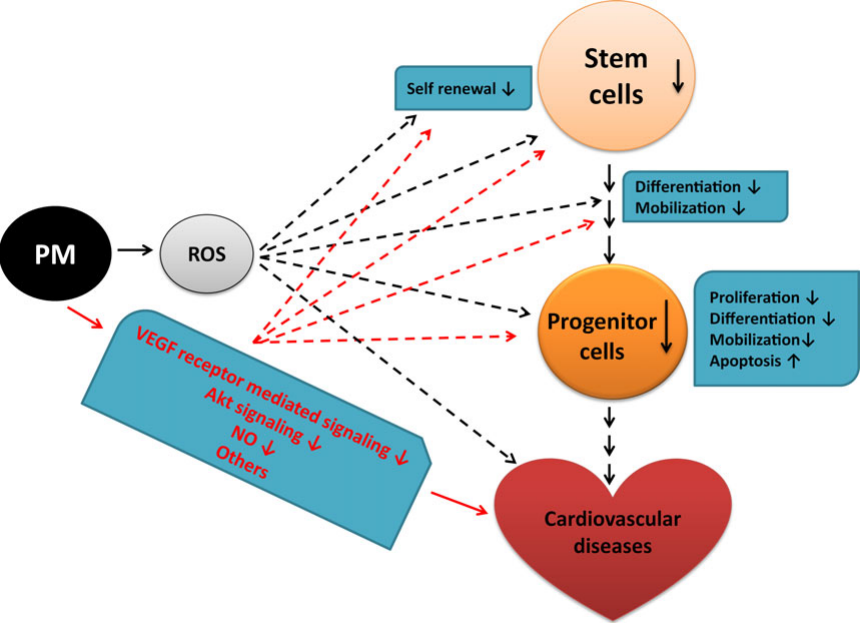
Illustration of potential mechanisms for the effect of PM exposure on cardiovascular system and progenitor/stem cells. PM exposure resulted in ROS formation that in turn could lead to the detrimental effects on cardiovascular system and impaired number and function of progenitor/stem cells. The mechanisms for decreased number and function of progenitor/stem cells following PM exposure might be because of the inhibition of self‐renewal, proliferation, survival (enhanced apoptosis), homing, mobilization, adhesion to extracellular matrices or differentiation. Blocking PM‐induced ROS formation might be an effective treatment option to attenuate or prevent the adverse effect of PM exposure on the progenitor/stem cells and cardiovascular system. Other mechanisms like reduction in VEGF receptor‐mediated signalling, decreased Akt signalling and nitric oxide level could be also important for the effect of PM exposure. PM: particulate matter; ROS: reactive oxygen species. ‐: diminish; ↓: decrease.

It is well known that EPCs play a critical role in vascular repair, angiogenesis and maintaining normal endothelial function 13, 14, 15, 16. Particulate matter exposure has been reported to significantly decrease the number and function of EPCs, and thus increase the risk of cardiovascular diseases and adverse cardiovascular events. In 2010, O'Toole *et al*. recruited 16 healthy college students from Provo, UT, in the United States to participate in their study 18. The city of Provo is located in a valley and the temperature inversion in the valley could lead to a temporary increase in the concentration of PM_2.5_ in the atmosphere. In this study, the investigators demonstrated that episodic exposure to PM_2.5_ induced reversible vascular injury, decreased circulating EPC (CD34^+^/CD31^+^/CD45^+^/CD133^+^) levels, enhanced platelet activation, and increased plasma level of nonalbumin protein *in vivo*. In the same year, Liberda *et al*. reported that inhalation of nickel nanoparticles could result in a decrease in tube formation and chemotaxis function of EPCs (CD34^+^/VEGF‐R2^+^/CD11b^−^) *in vitro* as well as a reduction in EPC number in murine BM *in vivo* 66. A study performed in China in 2013 also showed that PM_2.5_ exposure decreased the number of EPCs (CD34^+^/KDR^+^, CD34^+^/KDR^+^/CD45^−^ or CD34^+^/KDR^+^/CD133^+^) in circulation 67. This Chinese study was conducted in two large adjacent communities in Jinchang and Zhangye with comparable ambient concentrations of PM_2.5_. Jinchang was identified as a heavily Nickel‐polluted area because of its proximity to the second largest Nickel refinery in the world. Zhangye, 250 miles northwest and upwind from Jinchang, was selected to serve as a control community. A total of 60 healthy non‐smoking adult women residents were recruited in the study. It was observed that the circulating EPCs were significantly lower in the participants from Jinchang than those from Zhangye. Diesel exhaust particles were reported to reduce the number and function of EPCs with impaired stromal cell‐derived factor (SDF)‐1‐induced migratory capacity and neoangiogenesis both *in vivo* and *in vitro* in a murine model. Consistent with above observation, we recently reported that PM treatment significantly decreased murine‐circulating EPC population, promoted apoptosis of murine EPCs (CD34^+^/CD133^+^) in association with increased ROS production and serum TNF‐α and IL‐1β levels *in vivo* (Fig. 1) 19.

However, some studies demonstrated that circulating EPCs could be increased after PM_2.5‐10_ exposure. Brook *et al*. recruited 32 healthy non‐smoking adults (18–50 years old) in Dexter, a town in Michigan, United States, an area with coarse PM_2.5‐10_ exposures 68. Dexter is 410 km from major freeways and 460 km west of the Detroit metropolitan area. The study showed that increased number of EPCs (CD34^+^/CD133^+^/CD3^−^/CD79b^−^/CD56^−^) *in vivo* persisted for at least 20 hrs following brief inhalation of coarse PM_2.5‐10_. The mechanism was believed to be related to a systemic reaction to an acute ‘endothelial injury’ and/or a circulating EPCs response to sympathetic nervous system activation. Haberzettl *et al*. delineated that exposure to PM_2.5_ could increase murine EPC (Sca‐1^+^/Flk‐1^+^) levels in the BM by preventing their mobilization to the peripheral blood *via* inhibition of signalling events triggered by VEGF‐receptor stimulation based on *in vivo and ex vivo experiment* 17. This might also explain the decreased circulating EPCs as a result of the decreased mobilization of BM EPCs into the circulation.

PM_2.5_ exposure might also have significant impact on APCs as well. Adipose tissue progenitor cells (Lin^−^/CD34^+^/CD29^+^/Sca‐1^+^/CD24^+^) in brown adipose tissue are closely correlated with the normal functionality of brown adipose tissue and reduction in obesity 69. It has been shown that high level exposure to PM_2.5_ in murine early life is associated with decreased number of murine APCs and increased risk factor for the development of insulin resistance, adiposity and inflammation in association with ROS generation by NADPH oxidase *in vivo* 70. The effect of PM exposure on different progenitor cells and their function in both human and animals was summarized in Tables 1 and 2.

**Table 1:** The effect of particulate air pollution on human progenitor/stem cells

Authors	Key findings	Participants	Exposure time	Location
O'Toole *et al*.	PM_2.5_ exposure decreases circulating EPC level	18–25 years adults	3 months	Utah, US
Brook *et al*.	Brief PM inhalation could increase EPC number for at least 20 hrs	18–50 years adults	2 hrs	Michigan US
Niu *et al*.	Specific metals in PM_2.5_ may be responsible for decreased circulating EPC level	60–65 years women	12 months	China
Lin *et al*.	CS inhibits ESCs growth	ESCs	6–24 hrs	Lab
Talbot *et al*.	CS could lead to poor adhesion to extracellular matrices, diminished survival and proliferation and increased apoptosis of ESCs	ESCs	6–24 hrs	Lab
Liszewski *et al*.	Tobacco smoking impairs foetal development	ESCs	8–21 days	Lab
Zhou *et al*.	Smoking inhibits BMSC recruitment and differentiation	MSCs	1 month	Lab

PM: particulate matter; SCs: stem cell; ESCs: embryonic stem cells; MSCs: mesenchymal stem cells; HSC: haematopoietic stem cells; BM: bone marrow; BMSCs: bone marrow stem cells; CSC: cigarette smoke condensate.

**Table 2:** The effect of particulate air pollution on animal progenitor/stem cells

Authors	Key findings	Participants	Exposure time
Xu *et al*.	PM2.5 exposure induces oxidative stress	Mouse APCs	10 weeks
Liberda *et al*.	Ni nanoparticles result in reduced number and function of EPCs in bone marrow	Mouse EPCs	2–5 days
Haberzettl *et al*.	PM_2.5_ exposure increases EPC levels in the bone marrow by preventing mobilization *via* inhibition of VEGF‐receptor signalling	Mouse EPCs	18 months
Poss *et al*.	Diesel exhaust particles impair EPC number and function *in vivo* and *in vitro*	Mouse EPCs	3–6 weeks
Cui *et al*.	PM exposure significantly decreased circulating EPCs population due to increased apoptosis *via* ROS formation	Mouse EPCs	1 month
Cui *et al*.	PM suppresses BMSC *in vivo* proliferation *via* ROS formation	Mouse SCs	1 month
Huang *et al*.	CS induces oxidative stress, telomere shortening, and apoptosis	Mouse EPCs	1 month
Yauk *et al*.	CS leads to mutations of spermatogonial SCs	Mouse spermatogonial SCs	6–12 weeks
Huang *et al*.	Acute CS exposure causes cell death and reduces pluripotency, while chronic CS exposure leads to DNA damage and telomere shortening	Mouse ESCs	20 hrs–2 weeks
Lin *et al*.	CS impairs ESC function	Mouse ESCs	6–24 hrs
Albrecht *et al*.	Titanium dioxide in coal dust induces hyperplasia of Clara cells	Rat Clara cell	126–129 weeks
Izzotti *et al*.	CS could induce recruitment of undifferentiated SC into lung	Mouse MSCs	1–4 months
De Flora *et al*.	Same as above	Mouse MSCs	1 week–11 months
Khaldoyanidi *et al*.	Nicotine could impair the function of the haematopoiesis‐supportive stromal microenvironment, and interfere with SCs homing	Mouse HSCs	1–4 weeks
Zhou *et al*.	Smoking inhibits BMSC recruitment and differentiation	Mouse MSCs	1 month

PM: particulate matter; APCs: adipose tissue progenitor cells; SCs: stem cell; ESCs: embryonic stem cells; MSCs: mesenchymal stem cells; HSC: haematopoietic stem cells; BM: bone marrow; BMSCs: bone marrow stem cells; CSC: cigarette smoke condensate.

## PM and stem cells

Cell‐based therapy with progenitor cells and SCs appears to be a promising option for the regeneration and/or repair of damaged tissues in cardiovascular system 71, 72, 73, 74. Many types of SCs have been studies as the potential sources for cell‐based therapy including embryonic SCs (ESCs) 75, 76, neural SCs 77, cardiac SCs 78, 79, 80, 81, BM‐derived haematopoietic SCs (HSCs) 82, BM‐derived c‐kit^+^/Lin^−^ cells 83, 84, mesenchymal SCs (MSCs) 72, 85, 86, adipose‐derived SCs 87 and inducible pluripotent stem cells from somatic cells 88. Progenitor/SCs in circulating blood and in the vascular wall could serve as the endogenous pool of SCs to restore the structural and functional integrity of the vasculature through rapid repair of the endothelial cells and/or formation of new vessels after injuries 89. The progenitor/SCs residing in vascular intima, media and adventitia may participate in vascular repair and the formation of neointimal lesions in severely damaged vessels 90. Recently, a new type of SCs was identified in the murine arterial media, named multipotent vascular SCs, which could differentiate into neural cells and MSC‐like cells and subsequently differentiate into SMCs 91. In addition, abundant progenitor/SCs expressing Sca‐1 have been identified in the adventitia, which may contribute to endothelial regeneration and smooth muscle accumulation in the neointimal lesions 92.

The therapeutic efficacy of cell therapy for cardiovascular diseases is associated with a variety of factors including cell types, myocardial ischemia, cardiac dysfunction or their combination 93. The outcome of cell therapy with stem cells could be also related to the engraftment and survival of the cells transplanted into the target area such as an infarcted myocardial area. It is known that one of the major challenges for cell therapy with BMSCs is the low viability of the implanted cells with the loss of cells occurring mainly in the first few days after *in vivo* delivery 94. However, the mechanisms for the poor *in vivo* survival of the cells are complex, and have yet to be defined. It is believed that an acute inflammatory reaction with formation of various inflammatory factors including inducible nitric oxide synthase in the delivery site is a critical factor for the cell death in the first 24–72 hr period 94, 95, 96.

Unfortunately, there is very little data available in the area of PM_2.5_ and SCs. We recently found that PM exposure significantly decreased murine BMSCs population *in vivo*, defined as lineage negative/Sca‐1 positive (LS) and lineage negative/CD133 positive (Lin^−^/CD133^+^) cells, in association with increased ROS formation, decreased level of Akt phosphorylation and inhibition of *in vivo* proliferation of murine BMSCs without induction of apoptosis 20. We further demonstrated that PM‐induced ROS production was the major mechanism for decreased *in vivo* proliferation and population of murine BMSCs. Treating mice with antioxidant N‐acetylcysteine (NAC) or using a triple transgenic mouse line with overexpression of antioxidant enzyme network (AON) composed of superoxide dismutase (SOD)1, SOD3 and glutathione peroxidase‐1 with decreased *in vivo* ROS production significantly decreased murine BMSCs intracellular ROS level, partially reversed the suppression of p‐Akt expression, effectively reversed the inhibition of BMSCs proliferation rate and restored the BMSCs population in the mice with PM exposure *in vivo* (Fig. 1).

There are a variety of sources for PM exposure 97. Recent studies showed that the median concentration of PM_2.5_ in the smoking area (both indoor and outdoor) was significantly higher than in the control area 98, 99, 100. Thus, environmental tobacco smoke‐associated PM could be an important independent health hazard in addition to the well‐known toxic and carcinogenic compounds contained in cigarette smoking (CS). It has been reported that CS could significantly impair the number and function of various SCs including ESCs, spermatogonial SCs (SSCs) and Clara cell (SCs of the bronchiolar epithelium). Cigarette smoking could produce cytotoxic action on human ESCs (hESCs) and mouse ESCs (mESCs), induce oxidative stress, apoptosis and telomere shortening in ESCs *in vitro*, inhibit cell adhesion and growth *in vitro*, and compromise embryo development *in vivo* 101, 102, 103. In addition, CS might also induce mutation of SSCs gene and alterations in hESCs gene expression (especially those characteristic for mesoderm and ectoderm development) *in vivo* 104. Increased expression of Notch, Wnt or transforming growth factor‐β genes by smoking resulted in retention of the cells in pluripotent state *in vivo* 105. In addition, acute exposure of mESCs to CS or cadmium could cause immediate cell death, and decrease their pluripotency, while chronic exposure could lead to DNA damage and telomere shortening *in vivo* 106, 107. Coal dust exposure resulted in the disappearance of proliferating cell nuclear antigen in *rat Clara cell in vivo* 108. Although CS could recruit SCs into murine lung *in vivo* 109, 110, negative impact including interfering murine and human MSCs homing by targeting microvascular endothelial cells and differentiation into endometrial cells and blood vessel *ex vivo* were reported 111, 112. The detrimental effects of PM exposure and CS on SCs were summarized in Tables 1 and 2. However, it is important to differentiate the effect of PM exposure from that of other toxic and carcinogenic compounds in CS on SCs.

## Possible mechanisms for the effects of PM exposure on progenitor/stem cells

There is growing evidence that supports an important role of oxidative stress in response to air pollution in different organ systems 113. Reactive oxygen species could function as signalling molecules in PM_2.5_‐trigged autophagy in human epithelia A549 cells 114. Oxidative stress could be triggered by PM_2.5_ and result in alterations in mitochondrial gene expression in brown adipose tissue 115. Clinical studies suggested that ROS formation, oxidative stress and inflammation induced by PM_2.5_ exposure were closely related to paediatric asthma 116. A relationship has been observed between ambient PM_10_, oxidative burden and carotid intima‐media thickness (a change and indicator for subclinical atherosclerosis) 117. Studies, using a simulated respiratory tract lining fluid model with three major water soluble antioxidants (glutathione, urate and ascorbate) at physiological concentrations that served as the first‐line defence in the airway against the oxidative activity of PM, showed that PM could deplete the antioxidants 118. It was also demonstrated that a close relationship was present for ultrafine particles and NO_2_/NO_x_ 119.

Reactive oxygen species and oxidative stress are involved in EPCs dysfunction in many disease states including hyperlipidaemia, DM and CAD 13, 15, 44. It was observed that the functional impairment of human EPCs by diesel exhaust particles was associated with an increased superoxide production 120. We also observed that ROS production was significantly increased in the EPCs and BMSCs from the mice exposed to PM. Blockage of ROS formation using pharmacological agent NAC or transgenic model with overexpression of NOA effectively prevented PM‐induced decrease in the numbers of circulating EPCs and BMSCs. These data suggested that ROS formation was an important cause for decreased number of EPCs 19 and BMSCs following PM exposure (Fig. 1).

Particulate matter exposure was shown to suppress VEGF‐induced Akt activation and endothelial nitric oxide synthase (eNOS) phosphorylation in the aorta, and prevented VEGF/AMD3100‐induced mobilization of EPCs into the peripheral circulation without change in the plasma levels of human SDF‐1α and VEGF 17, 66. Second‐hand smoke exposure was also reported to block VEGF‐stimulated nitric oxide production 121. There are extensive and complex interactions between ROS and Akt pathway in both normal and cancer cells. We observed that PM exposure inhibited BMSC proliferation *via* ROS‐mediated mechanism(s) partially through suppression of Akt signalling. It is certainly possible that other pathways might also be affected by PM exposure. Future studies are needed to define the role of other pathways in the effect of PM exposure on BMSCs and progenitor cells.

## Clinical implications for PM‐induced detrimental effects on progenitor/stem cells in cardiovascular system

It is clear that exposure to PM increases the risk of cardiovascular diseases with ROS formation as the predominant mechanism. It could be ideal to avoid inhaling PM physically *via* wearing masks or using filters. However, fine PM such as PM_2.5_ is very difficult to be removed or isolated from the air because of their extremely small size, especially in those cities with severe air pollution. Moreover, PM_2.5_ widely exists in the environment and may carry ROS within gas phase 122 or water phase (aerosol) 123 into the lower respiratory tract to create an increased risk for adverse cardiovascular events.

Antioxidant enzyme and antioxidant supplementation have been examined for its impact on cardio‐respiratory effects of PM_2.5_ exposure. Animal studies have shown an increase in the levels of antioxidant gene expression in epithelial cells after exposure to diesel exhaust particles 124. It was reported that omega‐3 polyunsaturated fatty acid could attenuate the adverse effect of PM_2.5_ on heart rate variability 125. Antioxidant supplementation such as vitamin C and E was shown to have beneficial effects against human lung damage by air pollution 126. Antioxidant probucol could reduce CS‐induced impairment of neovascularization associated with improved function of EPCs 127. Inhibition of ROS accumulation/production or oxidative stress with pharmacological agents like NAC and SOD‐mimics, or overexpression of antioxidant enzymes like Hsp20 and SOD could reduce ROS accumulation in human MSCs, and attenuate oxidative cell damage in BMSCs *in vitro* 128, protect stem cells against ROS‐induced apoptosis *in vitro* 129, protect MSCs against cell death triggered by oxidative stress *in vitro* in association with enhanced Akt activation and increased secretion of growth factors (such as VEGF, fibroblast growth factor‐2, and insulin‐like growth factor 1) 130, increase the differentiation of EPCs into endothelial cells 131, inhibit cell senescence in HSCs in the BM 132 and restore the impaired self‐renewal potential and functional activity of HSCs with high ROS level 133. N‐acetylcysteine treatment also protected BMSCs against the toxic effect of low concentration ox‐LDL, and restored their endothelial differentiation potential impaired by ox‐LDL 134. Recently, we observed that after PM_≤4_ exposure, NAC or overexpression of AON could completely block intracellular ROS production in BMSCs, partially restore p‐Akt level, decrease serum TNF‐α and IL‐1β level, reduce EPCs apoptotic rate, effectively reversed the decreased proliferation rate of BMSCs and increased the BMSCs and EPCs number to normal level 19. Thus, inhibition of ROS production and oxidative stress might be an effective option to ameliorate PM‐induced detrimental effects on progenitor cells and SCs as well as cardiovascular system.

## Other considerations

There is no question that PM exposure has significant impact on the number and function of progenitor cells and stem cells. However, studies are needed to address a variety of important issues in this area including (but not limited to): (*i*) determining the size and active components of PM that are responsible for detrimental effect of PM exposure on progenitor cells and SCs, as well as cardiovascular diseases and related mechanism(s) since the size and components are critical to the action of PM 135; and (*ii*) defining the mode of actions (direct or indirect) for PM exposure on the progenitor cells and SCs.

The number and function of progenitor cells and SCs are associated with other factors and cells like monocytes and platelets through a wide range of cytokines and growth factors 136, 137, 138, 139. Both monocytes and platelets are important to cardiovascular physiology (like angiogenesis and haemostasis), and closely related to cardiovascular diseases like CAD. It is known that monocytes display certain plasticity and could function as pluripotent stem cells with regenerative capability, and produce a variety of cytokines and inflammatory factors 140, 141. Particulate matter exposure could exhibit its effects on progenitor cells and SCs through functional and/or structural modifications of monocytes and platelets. Indeed, PM exposure is able to significantly alter the function and responses of platelets both *in vitro* and *in vivo* including induction of Ca(2+) release, dense granule secretion and surface expression of platelet activation markers like P‐selectin expression, as well as aggregation, and change in the mean platelet volume 142, 143, 144, 145, 146. Particulate matter exposure/treatment has been shown to modify the function of monocytes significantly including inflammatory response and cytokine production, recruitment and mobilization, and transcriptional and translational modulations of gene expressions in monocytes 135, 136, 147, 148, 149, 150, 151, 152. Further studies are needed to determine the complex relations between PM exposure, monocyte and platelet function, and progenitor cells/SCs.

## Conclusion

In this review, we discussed the adverse effects of PM exposure on cardiovascular diseases with specific effort on PM‐induced detrimental impact on progenitor/SCs. Indeed, PM exposure correlated with the reduction in life expectancy primarily *via* cardiovascular diseases, and the resultant abnormality in the number and function of progenitor/SCs might play an important role in cardiovascular diseases related to PM exposure (Fig. 1). However, there are lots of questions that need to be addressed on PM‐induced structural and functional impairment on progenitor/SCs. For example, does PM affect the differentiation potential of BMSCs and how? Does PM affect other SCs and how? All these questions require further studies. Although prevention of ROS formation and oxidative stress might be an effective way to attenuate PM‐induced deleterious effects on progenitor/SCs, we believe that other mechanisms may be also important for the effect of PM exposure, which merit further investigations.

## Conflicts of interest

No conflict of interest.
